# Supplementary material for: Detecting rhythmic spiking through the power spectra of point process model residuals
Source: bioRxiv. 2024 Mar 25:2023.09.08.556120. Preprint. [Version 2] doi: 10.1101/2023.09.08.556120 (PMC10996479; doi:10.1101/2023.09.08.556120)
Supplement: 1 [file NIHPP2023.09.08.556120V2-supplement-1.pdf]

bioRxiv preprint doi: https://doi.org/10.1101/2023.09.08.556120; this version posted March 25, 2024. The copyright holder for this preprint (which was not certified by peer review) is the author/funder, who has granted bioRxiv a license to display the preprint in perpetuity. It is made available under aCC-BY 4.0 International license.

# Supporting Information

## Supporting Figures

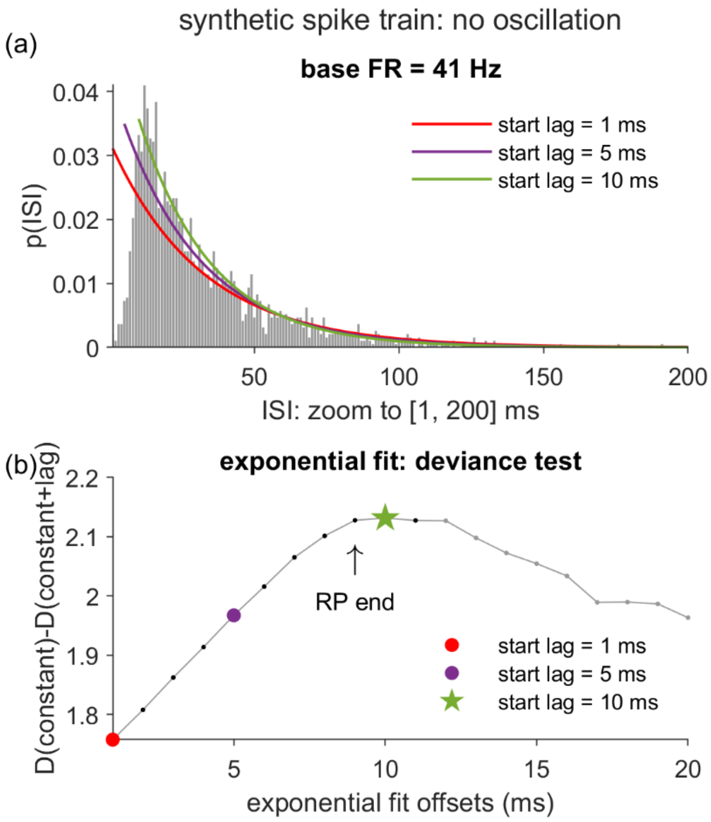


Fig. S1. Estimation of recovery period duration: No oscillation example**.** (a) Illustration of the procedure for obtaining an estimate of the RP duration ($\hat{n}_r$), as applied to a synthetic spike train with no oscillation (modulation strength $m$ = 0). A series of right-shifted exponential curves are fit to the ISI distribution, left-anchored to starting positions advanced in 1 ms steps (with 3 sample iterations highlighted in the figure). (b) Plot of the deviance difference statistic, ΔD, as a function of the first 20 starting positions of the exponential fits. D(constant), D(constant+lag) = deviance measures for the intercept-only and intercept+exponential curve models, respectively. ΔD tracks the goodness of fit contributed by the exponential curve. The $\hat{n}_r$ estimate is set equal to the post-spike lag immediately preceding the first local maximum in the ΔD plot.

bioRxiv preprint doi: https://doi.org/10.1101/2023.09.08.556120; this version posted March 25, 2024. The copyright holder for this preprint (which was not certified by peer review) is the author/funder, who has granted bioRxiv a license to display the preprint in perpetuity. It is made available under aCC-BY 4.0 International license.

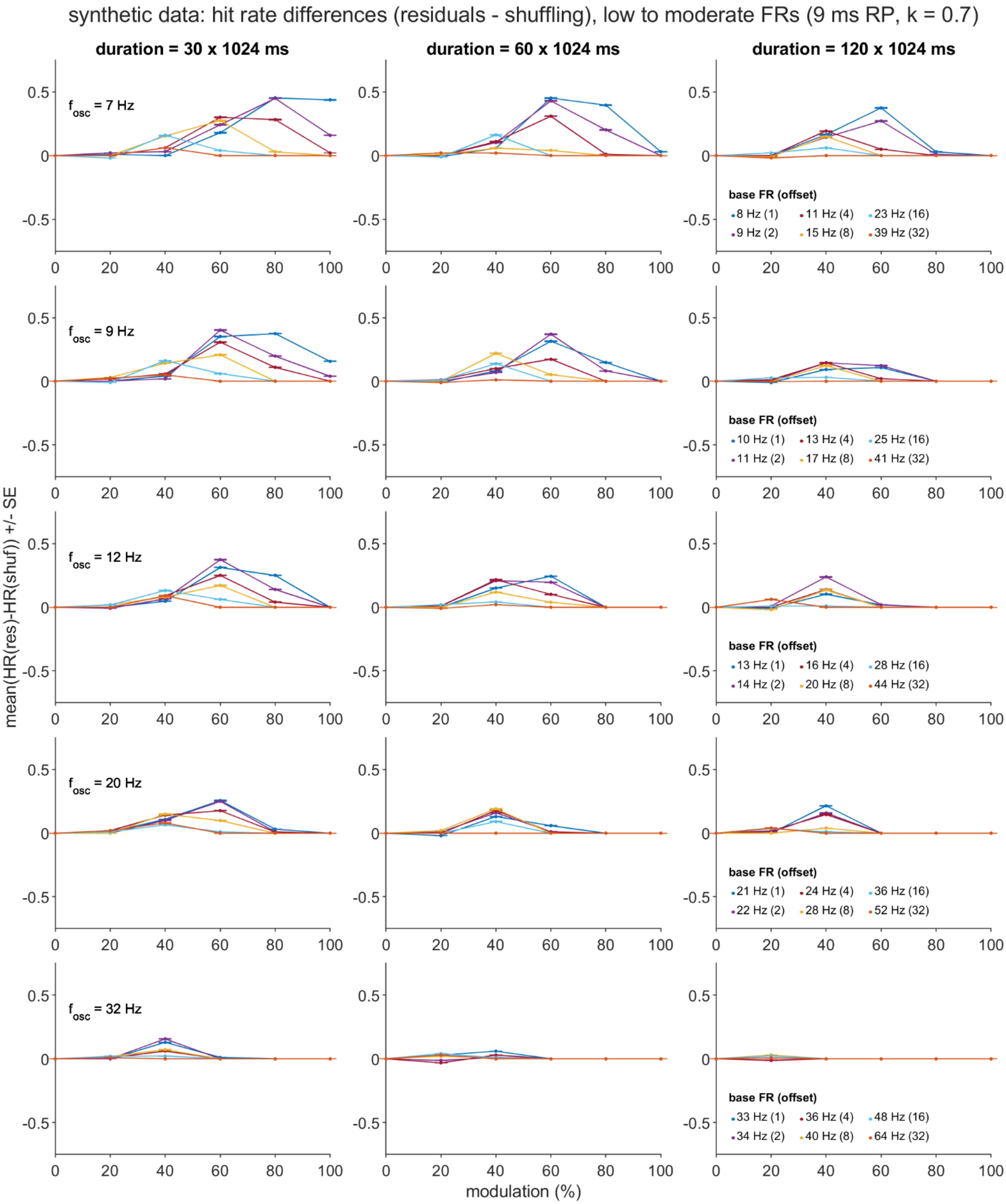

bioRxiv preprint doi: https://doi.org/10.1101/2023.09.08.556120; this version posted March 25, 2024. The copyright holder for this preprint (which was not certified by peer review) is the author/funder, who has granted bioRxiv a license to display the preprint in perpetuity. It is made available under aCC-BY 4.0 International license.

Fig. S2. Residuals (res) - shuffling (shuf) difference in hit rates ($D_{HR}$) over the varied parameters of the primary synthetic dataset. Means and standard errors (SE) reflect summaries over the hit rates (HR) computed for each of 1000 subsamples of the original, primary synthetic dataset, which was generated using low-to-moderate firing rates (FR) and a 9 ms relative recovery period (RP; $k$ = steepness parameter). See Methods and the Figure 4 caption for details regarding the definition of a hit and the subsampling procedure. Plots depict all 540 unique combinations of oscillation frequency ($f_{osc}$, rows), simulation duration ($T$, columns), oscillation modulation strength ($m$, $x$ axes) and base FR - oscillation frequency offset ($p_{base\_offset}$, lines).

bioRxiv preprint doi: https://doi.org/10.1101/2023.09.08.556120; this version posted March 25, 2024. The copyright holder for this preprint (which was not certified by peer review) is the author/funder, who has granted bioRxiv a license to display the preprint in perpetuity. It is made available under aCC-BY 4.0 International license.

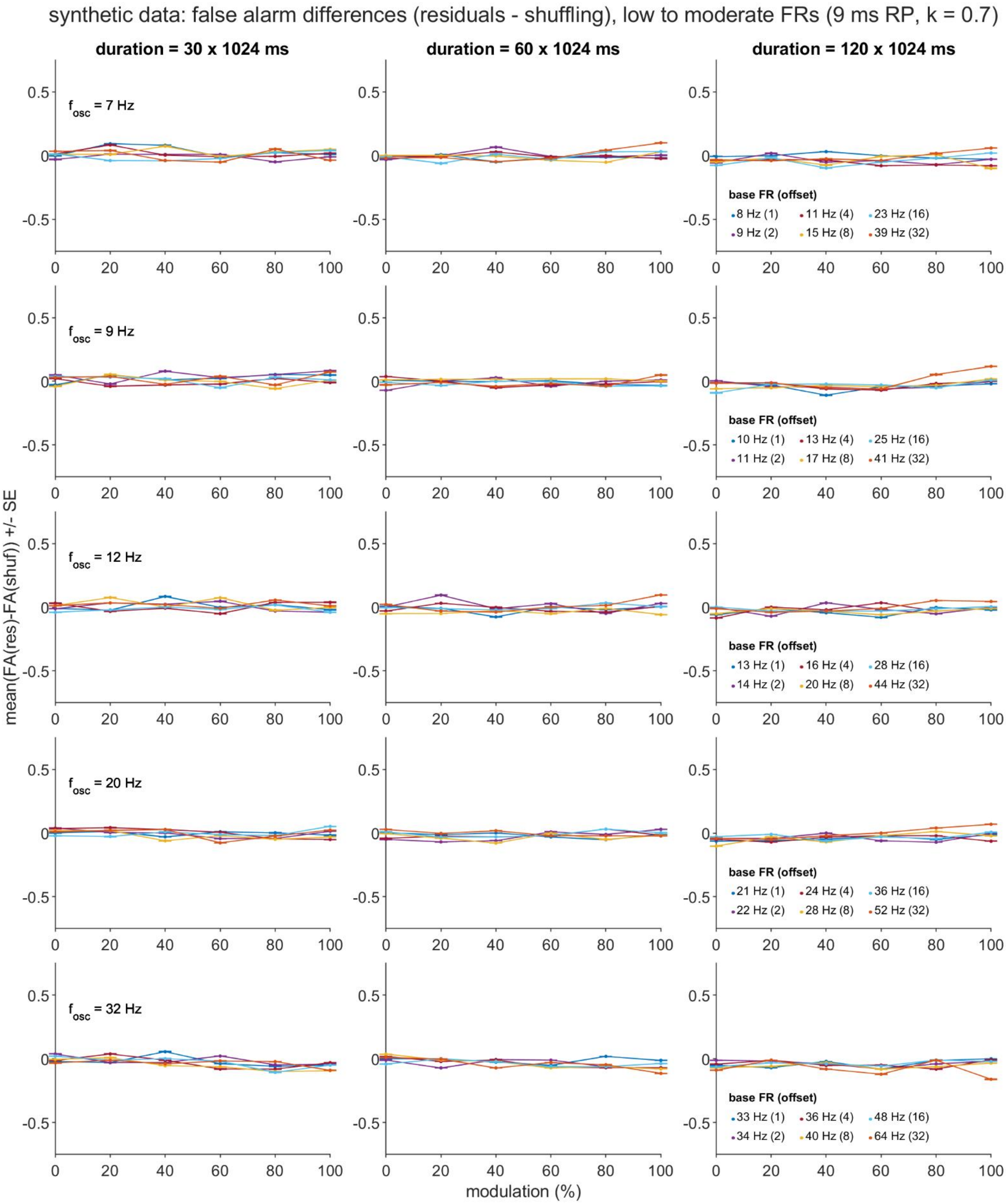

bioRxiv preprint doi: https://doi.org/10.1101/2023.09.08.556120; this version posted March 25, 2024. The copyright holder for this preprint (which was not certified by peer review) is the author/funder, who has granted bioRxiv a license to display the preprint in perpetuity. It is made available under aCC-BY 4.0 International license.

Fig. S3. Residuals - shuffling difference in false alarm rates ($D_{FA}$) over the varied parameters of the primary synthetic dataset. Means and standard errors reflect summaries over the false alarm rates (FA) computed for each of 1000 subsamples of the original dataset. See the Figure 4 caption for details regarding the definition of a false alarm. Abbreviations, plotting conventions, and the subsampling procedure follow from those described for Fig. S2 and Fig. 4.

bioRxiv preprint doi: https://doi.org/10.1101/2023.09.08.556120; this version posted March 25, 2024. The copyright holder for this preprint (which was not certified by peer review) is the author/funder, who has granted bioRxiv a license to display the preprint in perpetuity. It is made available under aCC-BY 4.0 International license.

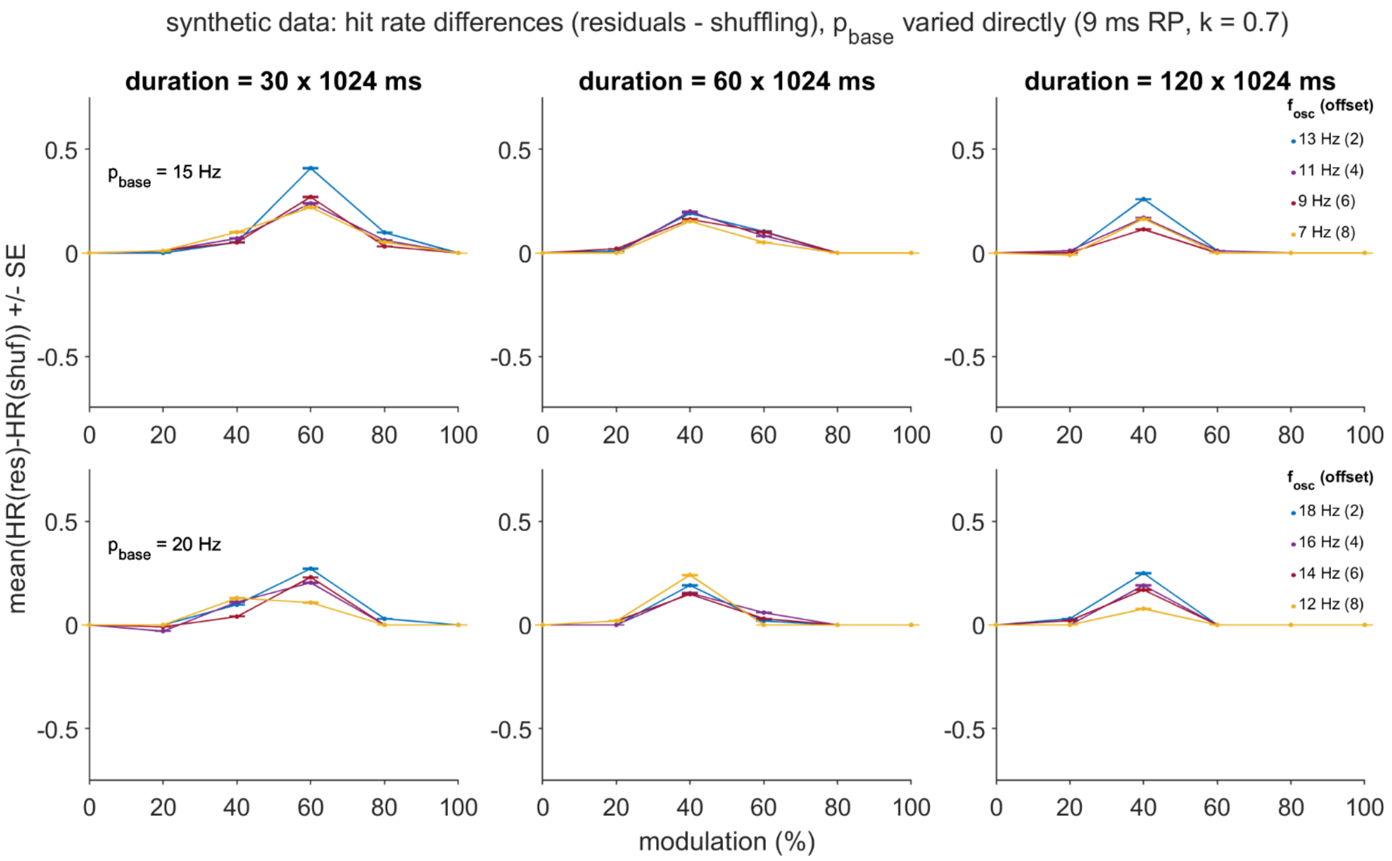


Fig. S4. **Residuals - shuffling difference in hit rates over the varied parameters of the dataset formed with direct base firing rate manipulation.** Means and standard errors reflect summaries over the hit rates computed for each of 1000 subsamples of a dataset in which the $p_{base}$ (i.e., base FR) and $p_{base\_offset}$ parameters were varied directly (as opposed to $p_{base\_offset}$ and $f_{osc}$). Plots depicts all 144 unique combinations of $p_{base}$ (rows), simulation duration ($T$, columns), oscillation modulation strength ($m$, $x$ axes), and oscillation frequency ($f_{osc}$, lines). All other abbreviations follow from those described for Fig. S2. See the Methods for a description of the subsampling procedure for this dataset.

bioRxiv preprint doi: https://doi.org/10.1101/2023.09.08.556120; this version posted March 25, 2024. The copyright holder for this preprint (which was not certified by peer review) is the author/funder, who has granted bioRxiv a license to display the preprint in perpetuity. It is made available under aCC-BY 4.0 International license.

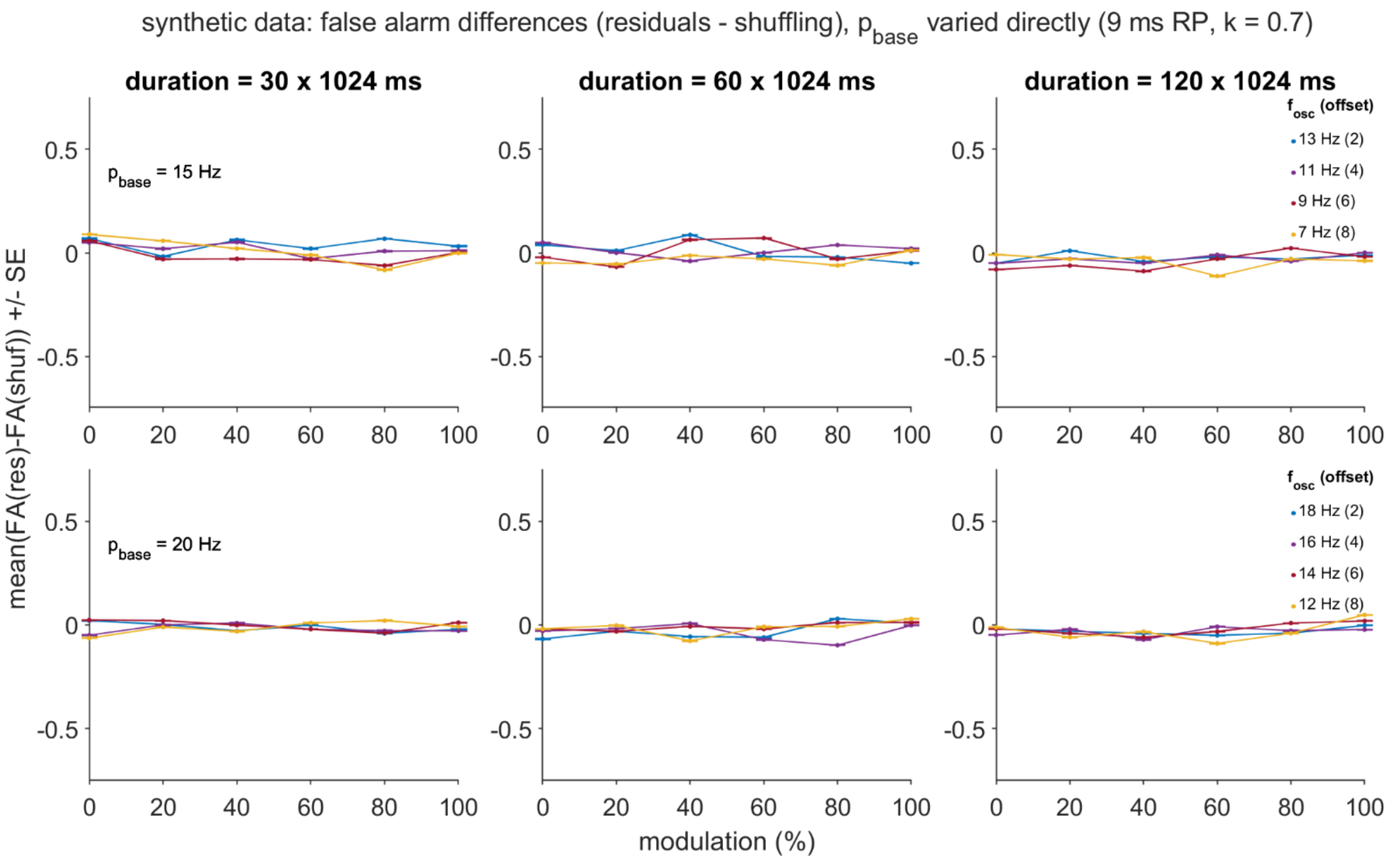


Fig. S5. Residuals - shuffling difference in false alarm rates over the varied parameters of the dataset formed with direct base firing rate manipulation. Means and standard errors reflect summaries over the false alarm rates computed for each of 1000 subsamples of a dataset in which the $p_{base}$ and $p_{base\_offset}$ parameters were varied directly. All other abbreviations, the plotting conventions, and the subsampling procedure follow from those described for Fig. S3-S4.

bioRxiv preprint doi: https://doi.org/10.1101/2023.09.08.556120; this version posted March 25, 2024. The copyright holder for this preprint (which was not certified by peer review) is the author/funder, who has granted bioRxiv a license to display the preprint in perpetuity. It is made available under aCC-BY 4.0 International license.

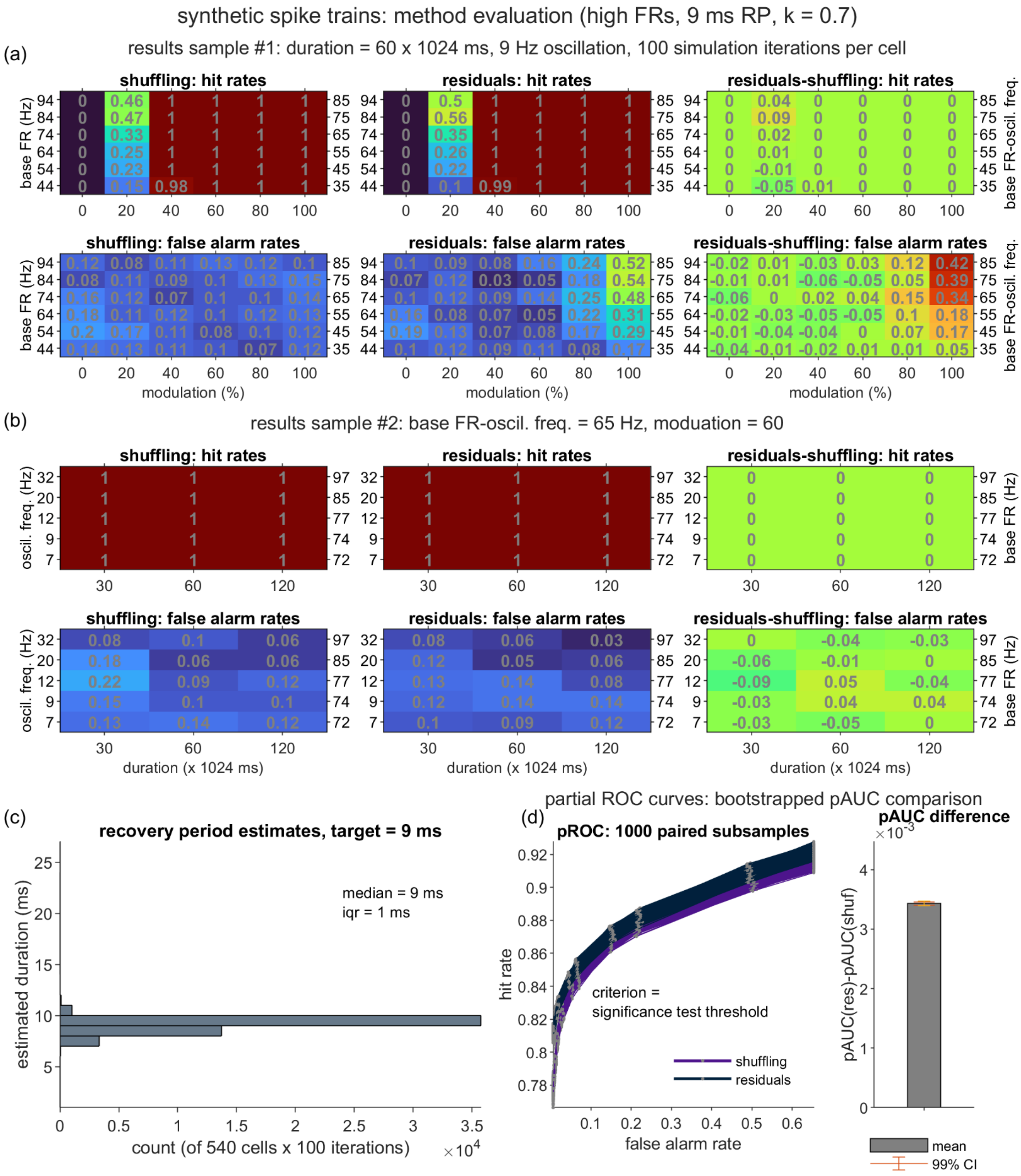

bioRxiv preprint doi: https://doi.org/10.1101/2023.09.08.556120; this version posted March 25, 2024. The copyright holder for this preprint (which was not certified by peer review) is the author/funder, who has granted bioRxiv a license to display the preprint in perpetuity. It is made available under aCC-BY 4.0 International license.

Fig. S6. Performance of the shuffling and residuals methods over a synthetic dataset of high firing rate (FR) spike trains. Panels (a)-(d): Plotting conventions, hit and false alarm definitions, and analysis procedures are identical to those described for the primary dataset depicted in Fig. 4. Relative to the primary dataset, this high FR dataset differed in the use of greater $p_{base\_offset}$ values (see "base FR - oscil. freq." tick labels in Panel (a)).

bioRxiv preprint doi: https://doi.org/10.1101/2023.09.08.556120; this version posted March 25, 2024. The copyright holder for this preprint (which was not certified by peer review) is the author/funder, who has granted bioRxiv a license to display the preprint in perpetuity. It is made available under aCC-BY 4.0 International license.

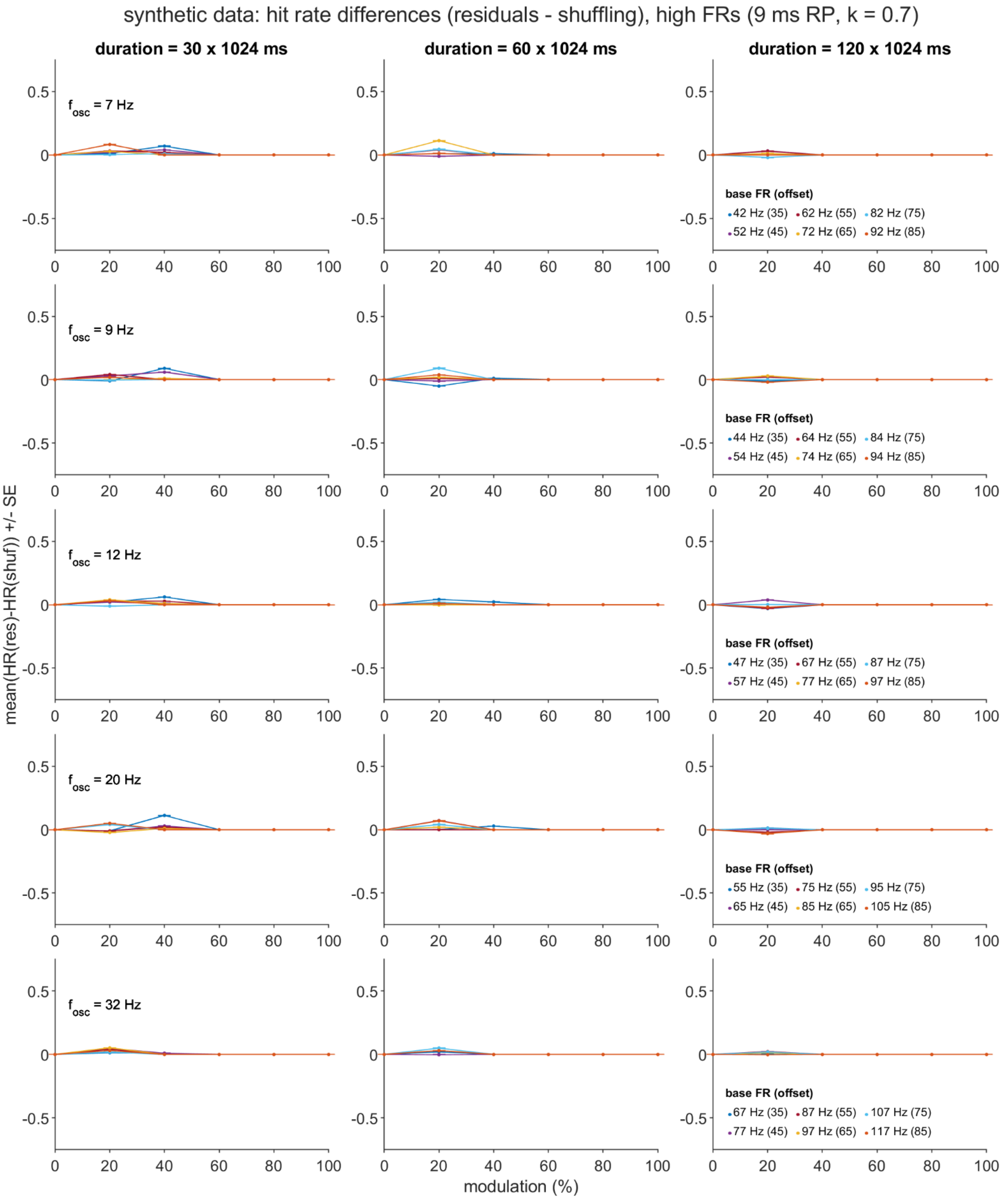

bioRxiv preprint doi: https://doi.org/10.1101/2023.09.08.556120; this version posted March 25, 2024. The copyright holder for this preprint (which was not certified by peer review) is the author/funder, who has granted bioRxiv a license to display the preprint in perpetuity. It is made available under aCC-BY 4.0 International license.

Fig. S7. Residuals - shuffling difference in hit rates over the varied parameters of the dataset of high firing rate spike trains. Means and standard errors reflect summaries over the hit rates computed for each of 1000 subsamples of the original, high FR dataset (see Fig. S6 for details). Abbreviations and plotting conventions follow from those described for Fig. S2.

bioRxiv preprint doi: https://doi.org/10.1101/2023.09.08.556120; this version posted March 25, 2024. The copyright holder for this preprint (which was not certified by peer review) is the author/funder, who has granted bioRxiv a license to display the preprint in perpetuity. It is made available under aCC-BY 4.0 International license.

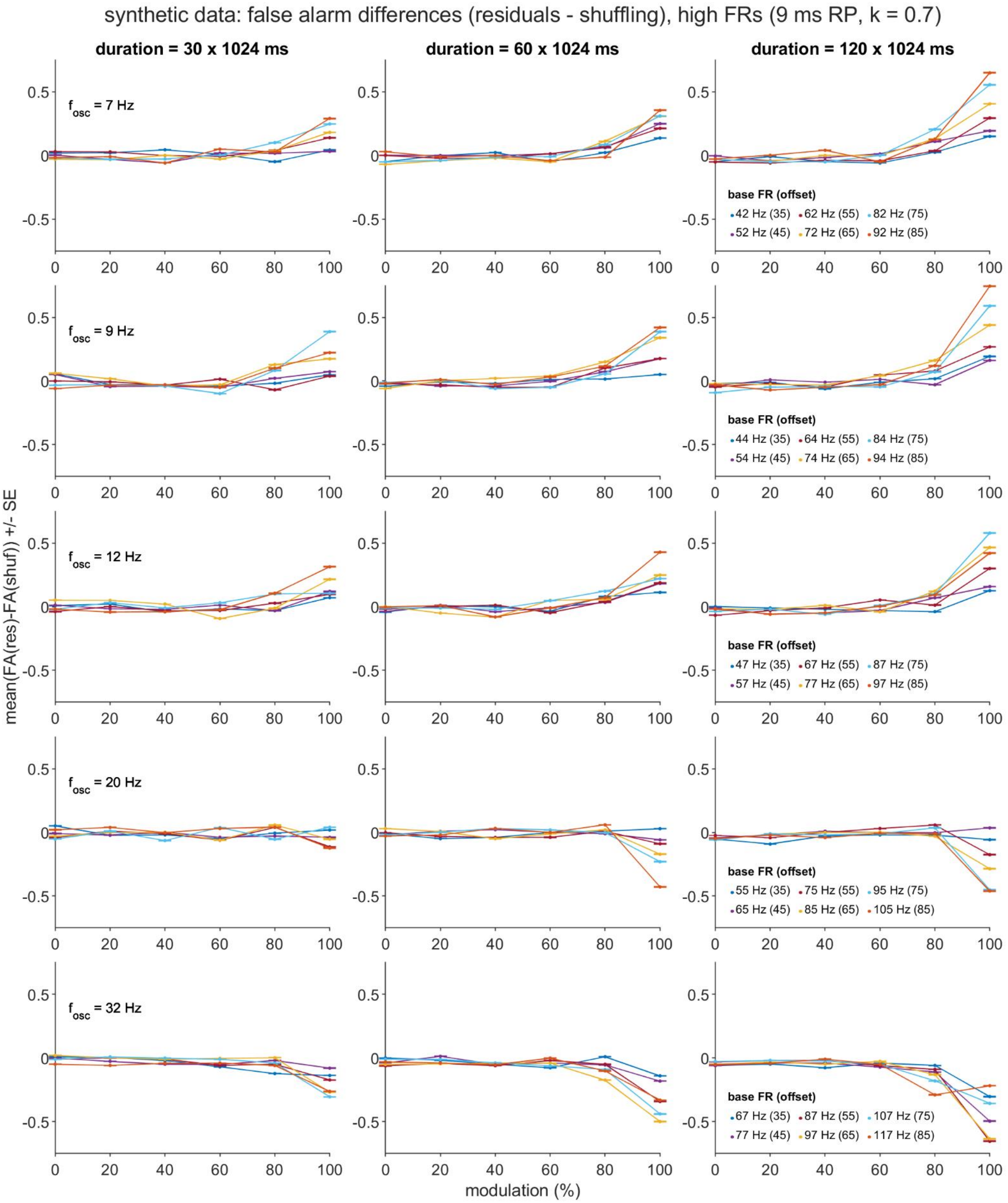

bioRxiv preprint doi: https://doi.org/10.1101/2023.09.08.556120; this version posted March 25, 2024. The copyright holder for this preprint (which was not certified by peer review) is the author/funder, who has granted bioRxiv a license to display the preprint in perpetuity. It is made available under aCC-BY 4.0 International license.

Fig. S8. Residuals - shuffling difference in false alarms over the varied parameters of the dataset of high firing rate spike trains. Means and standard errors reflect summaries over the false alarm rates computed for each of 1000 subsamples of the original, high FR dataset (see Fig. S6 for details). Abbreviations and plotting conventions follow from those described for Fig. S3.

bioRxiv preprint doi: https://doi.org/10.1101/2023.09.08.556120; this version posted March 25, 2024. The copyright holder for this preprint (which was not certified by peer review) is the author/funder, who has granted bioRxiv a license to display the preprint in perpetuity. It is made available under aCC-BY 4.0 International license.

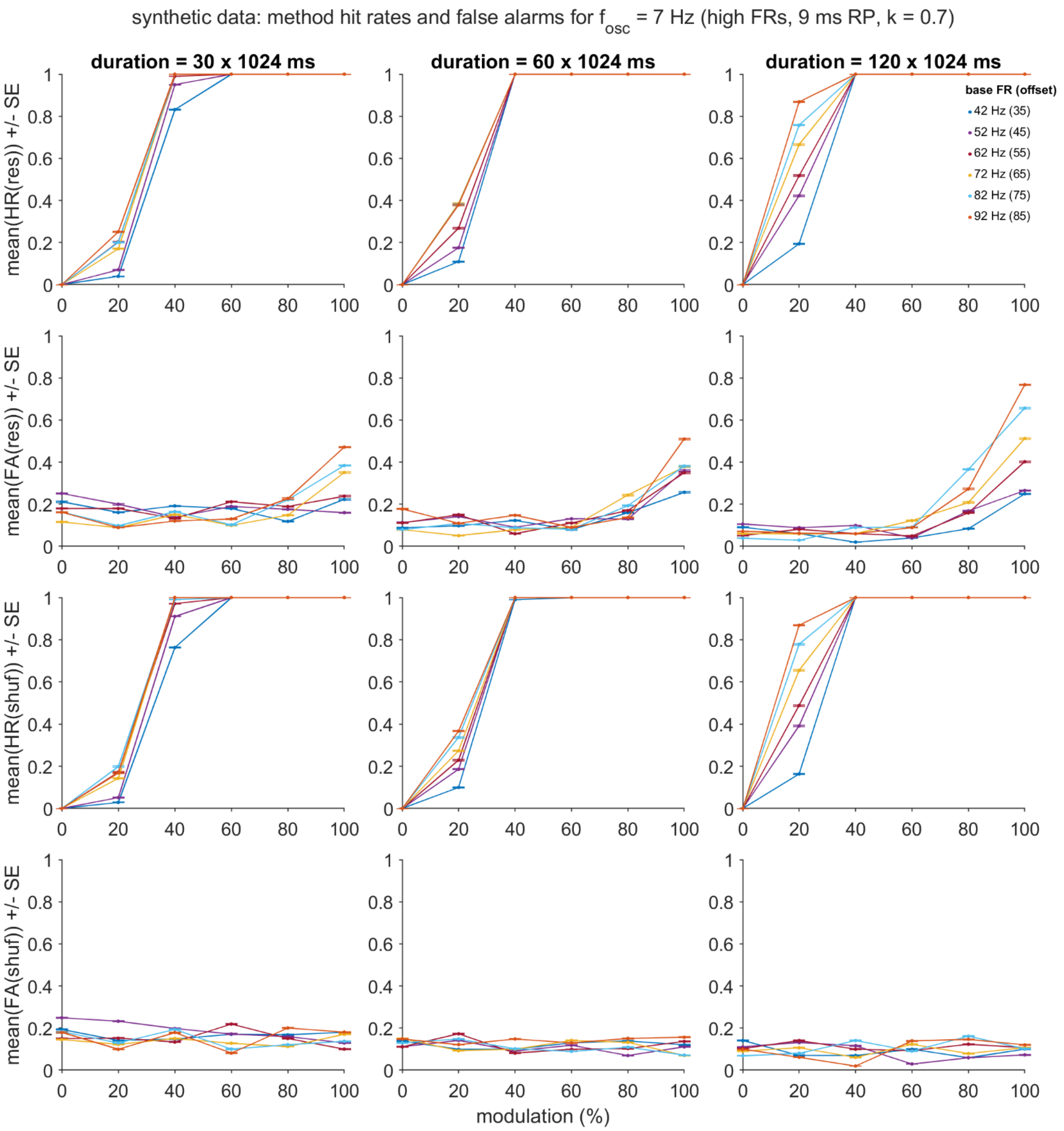


Fig. S9. Residuals and shuffling hit and false alarm rates for the dataset of high firing rate spike trains, with the oscillation frequency ($f_{osc}$) fixed at 7 Hz. Means and standard errors reflect summaries over the hit and false alarm rates computed for each of 1000 subsamples of the original, high FR dataset. Plots depict all 108 unique combinations of simulation duration ($T$, columns), oscillation modulation strength ($m$, $x$ axes) and base FR - oscillation frequency offset ($p_{base\_offset}$, lines) for the cases in which $f_{osc}$ = 7 Hz. Abbreviations follow from those described for Figs. S2-S3.

bioRxiv preprint doi: https://doi.org/10.1101/2023.09.08.556120; this version posted March 25, 2024. The copyright holder for this preprint (which was not certified by peer review) is the author/funder, who has granted bioRxiv a license to display the preprint in perpetuity. It is made available under aCC-BY 4.0 International license.

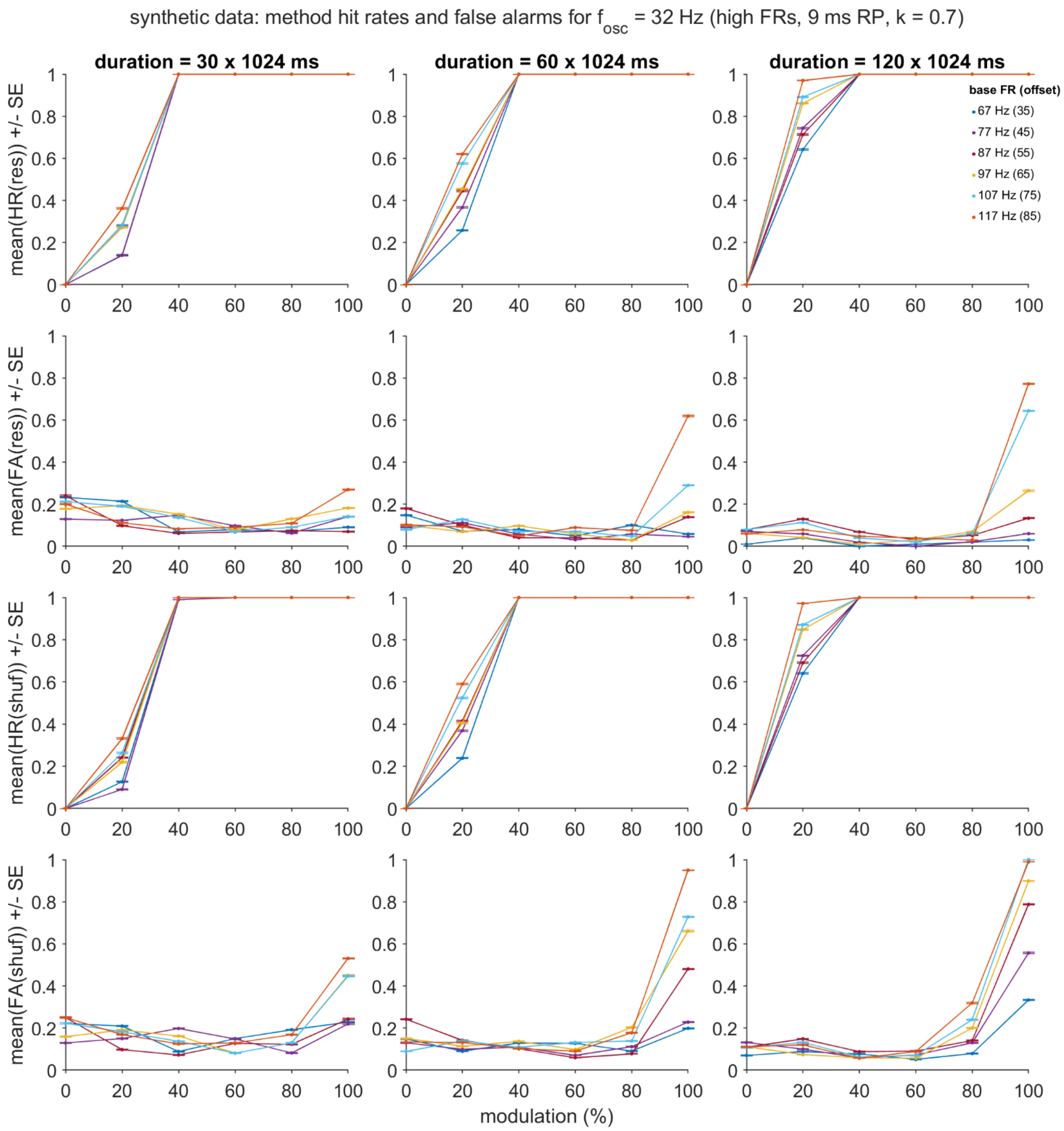


Fig. S10. Residuals and shuffling hit and false alarm rates for the dataset of high firing rate spike trains, with the oscillation frequency ($f_{osc}$) fixed at 32 Hz. Abbreviations and plotting conventions follow from those described for Fig. S2-S3 and S9.

bioRxiv preprint doi: https://doi.org/10.1101/2023.09.08.556120; this version posted March 25, 2024. The copyright holder for this preprint (which was not certified by peer review) is the author/funder, who has granted bioRxiv a license to display the preprint in perpetuity. It is made available under aCC-BY 4.0 International license.

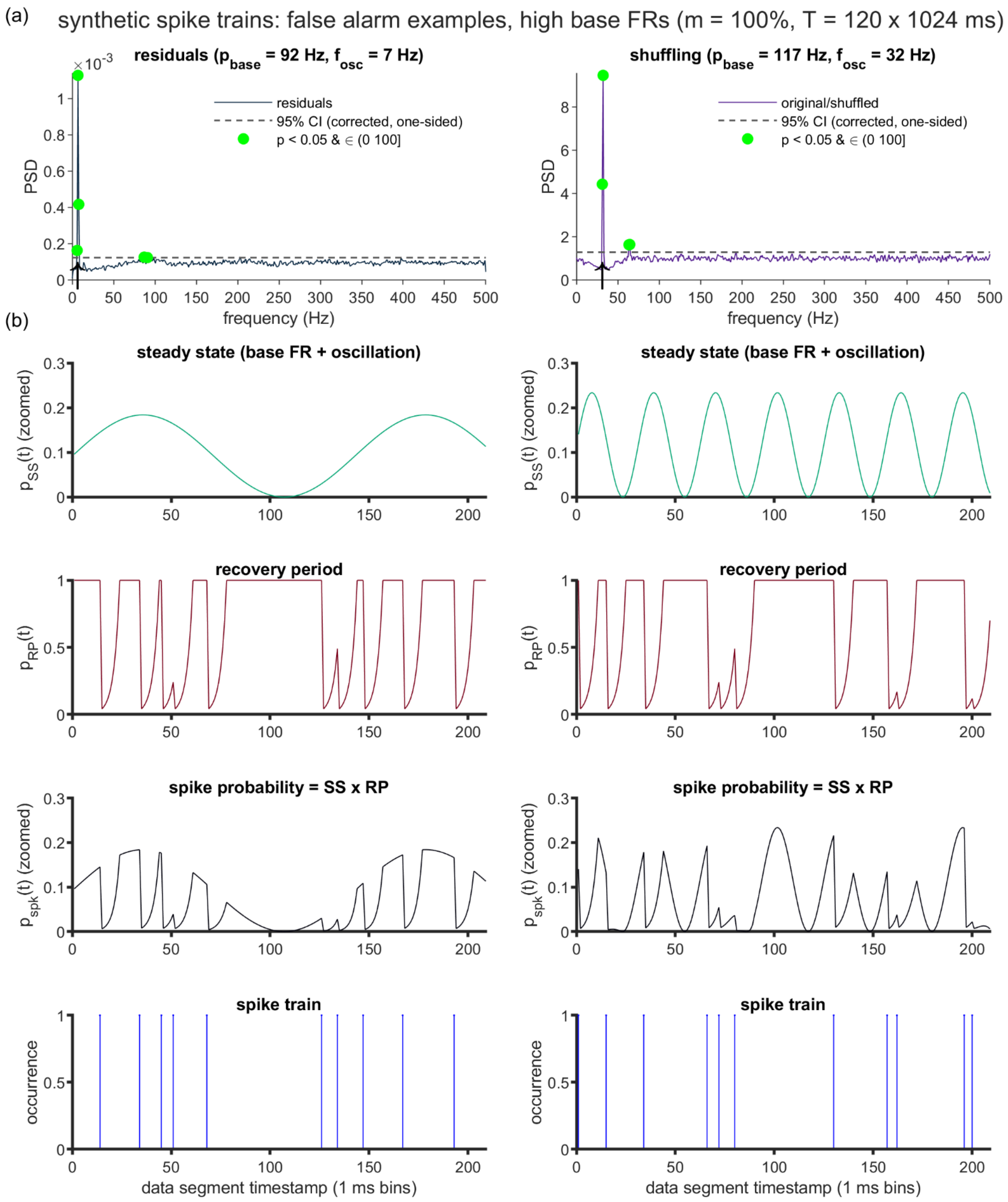

bioRxiv preprint doi: https://doi.org/10.1101/2023.09.08.556120; this version posted March 25, 2024. The copyright holder for this preprint (which was not certified by peer review) is the author/funder, who has granted bioRxiv a license to display the preprint in perpetuity. It is made available under aCC-BY 4.0 International license.

Fig. S11. Example power spectra and spike train segments for high firing rate cases that produced pronounced false alarms following residuals or shuffling correction. Two synthetic spike trains were generated to illustrate conditions that yielded especially high false alarm rates under residuals and shuffling correction, respectively. Both spike trains utilized the maximal modulation strength ($m$ = 1.0), duration ($T$ = 120 x 1024 ms), and base FR - oscillation frequency offset ($p_{base\_offset}$ = 85 Hz) settings from the original high FR dataset, and the default RP parameters (duration $n_r$ = 9 ms, steepness $k$ = 0.7). Oscillation frequency was set at either the lowest setting from the full high FR dataset (7 Hz, to illustrate residuals FAs) or the highest setting (32 Hz, to illustrate shuffling FAs). (a) Corrected power spectral density (PSD) functions generated by the residuals method (left) and shuffling method (right). Statistical testing and plotting conventions follow from those described for Fig. 2-3. (b) Illustration of the initial 209 ms of the synthetic spike trains (fourth row) from which the PSDs in (a) were computed, and the components of the rate function that governed their generation (first-third rows; see Methods, Eq. 1 for full details). Term definitions: $p_{ss}$(t) = steady state spiking probability, $p_{RP}$(t) = recovery period spiking probability, $p_{spk}$(t) = spike probability, reflecting the product of the steady state and recovery period components.

bioRxiv preprint doi: https://doi.org/10.1101/2023.09.08.556120; this version posted March 25, 2024. The copyright holder for this preprint (which was not certified by peer review) is the author/funder, who has granted bioRxiv a license to display the preprint in perpetuity. It is made available under aCC-BY 4.0 International license.

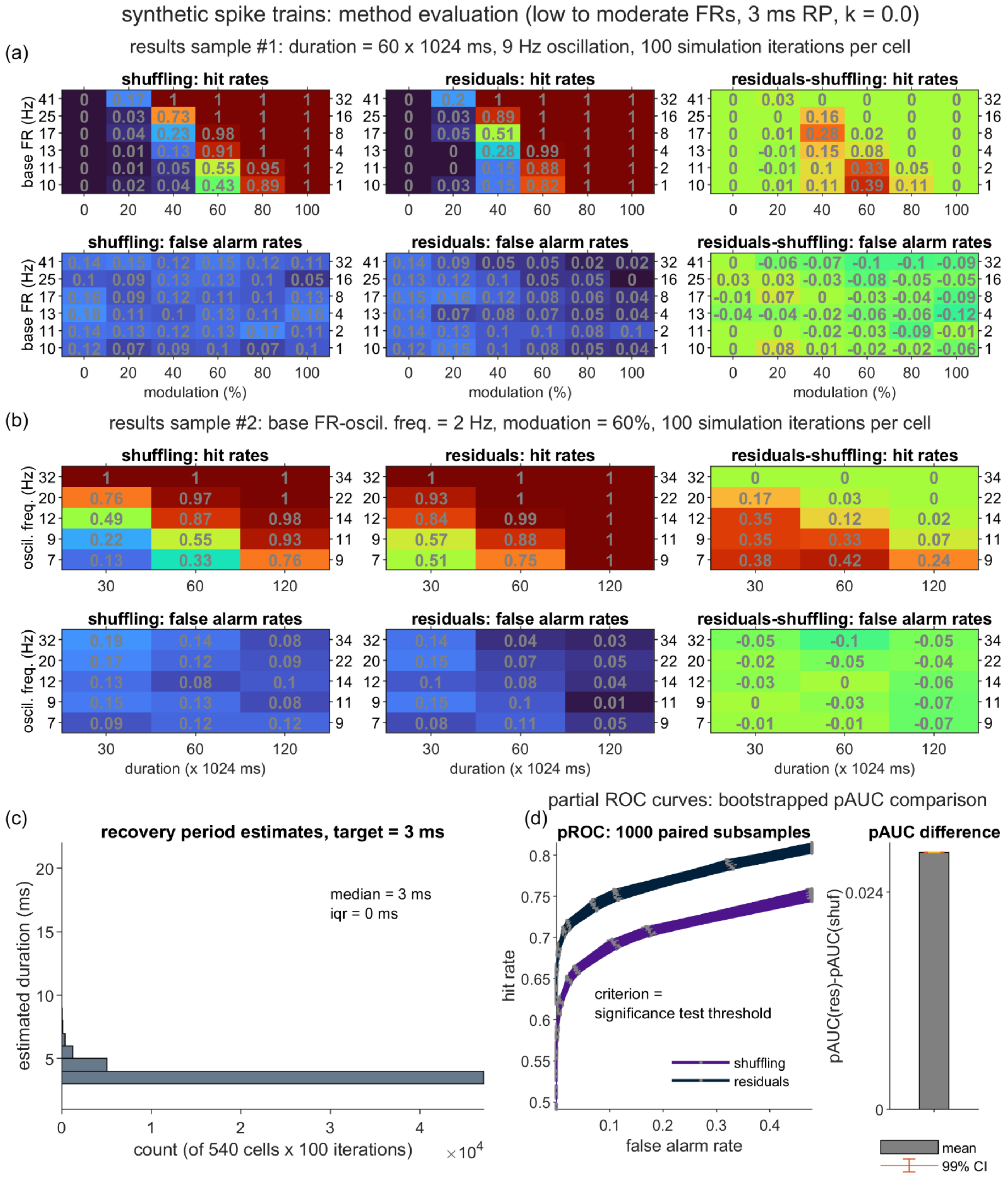

bioRxiv preprint doi: https://doi.org/10.1101/2023.09.08.556120; this version posted March 25, 2024. The copyright holder for this preprint (which was not certified by peer review) is the author/funder, who has granted bioRxiv a license to display the preprint in perpetuity. It is made available under aCC-BY 4.0 International license.

Fig. S12. Performance of the shuffling and residuals methods over a synthetic dataset generated using a short, absolute recovery period. Panels (a)-(d): Plotting conventions, hit and false alarm definitions, and analysis procedures are identical to those described for the primary dataset depicted in Fig. 4. Relative to the primary dataset, this secondary dataset differed in the use of an absolute, as opposed to relative RP ($k = 0$), and a shorter RP duration ($nr = 3$ ms).

bioRxiv preprint doi: https://doi.org/10.1101/2023.09.08.556120; this version posted March 25, 2024. The copyright holder for this preprint (which was not certified by peer review) is the author/funder, who has granted bioRxiv a license to display the preprint in perpetuity. It is made available under aCC-BY 4.0 International license.

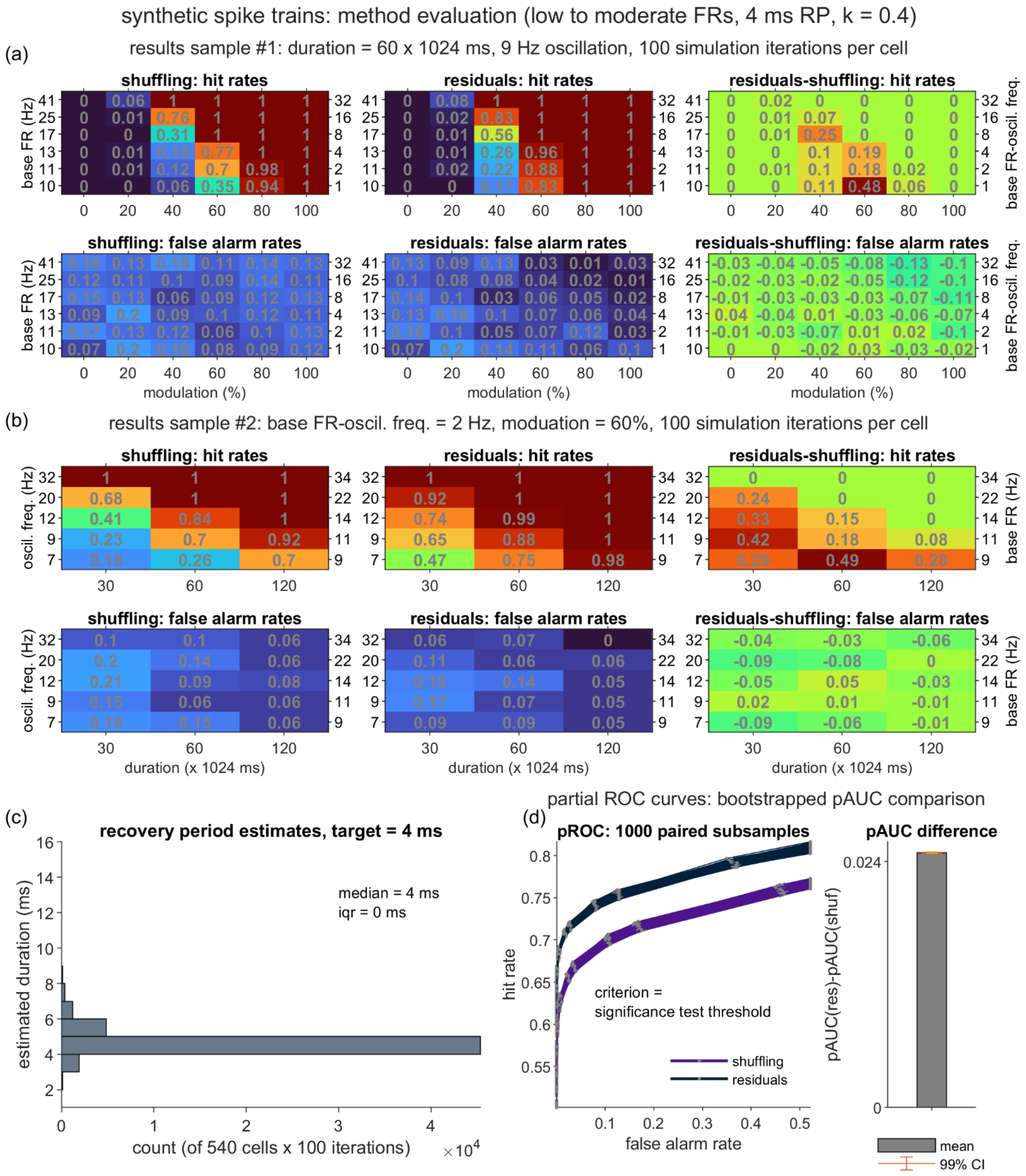

bioRxiv preprint doi: https://doi.org/10.1101/2023.09.08.556120; this version posted March 25, 2024. The copyright holder for this preprint (which was not certified by peer review) is the author/funder, who has granted bioRxiv a license to display the preprint in perpetuity. It is made available under aCC-BY 4.0 International license.

Fig. S13. Performance of the shuffling and residuals methods over a synthetic dataset generated using a shortened relative recovery period. Panels (a)-(d): Plotting conventions, hit and false alarm definitions, and analysis procedures are identical to those described for the primary dataset depicted in Fig. 4. Relative to the primary dataset, this secondary dataset differed in the use of a shortened and steeper relative RP ($n_r$ = 4 ms, $k$ = 0.4).

bioRxiv preprint doi: https://doi.org/10.1101/2023.09.08.556120; this version posted March 25, 2024. The copyright holder for this preprint (which was not certified by peer review) is the author/funder, who has granted bioRxiv a license to display the preprint in perpetuity. It is made available under aCC-BY 4.0 International license.

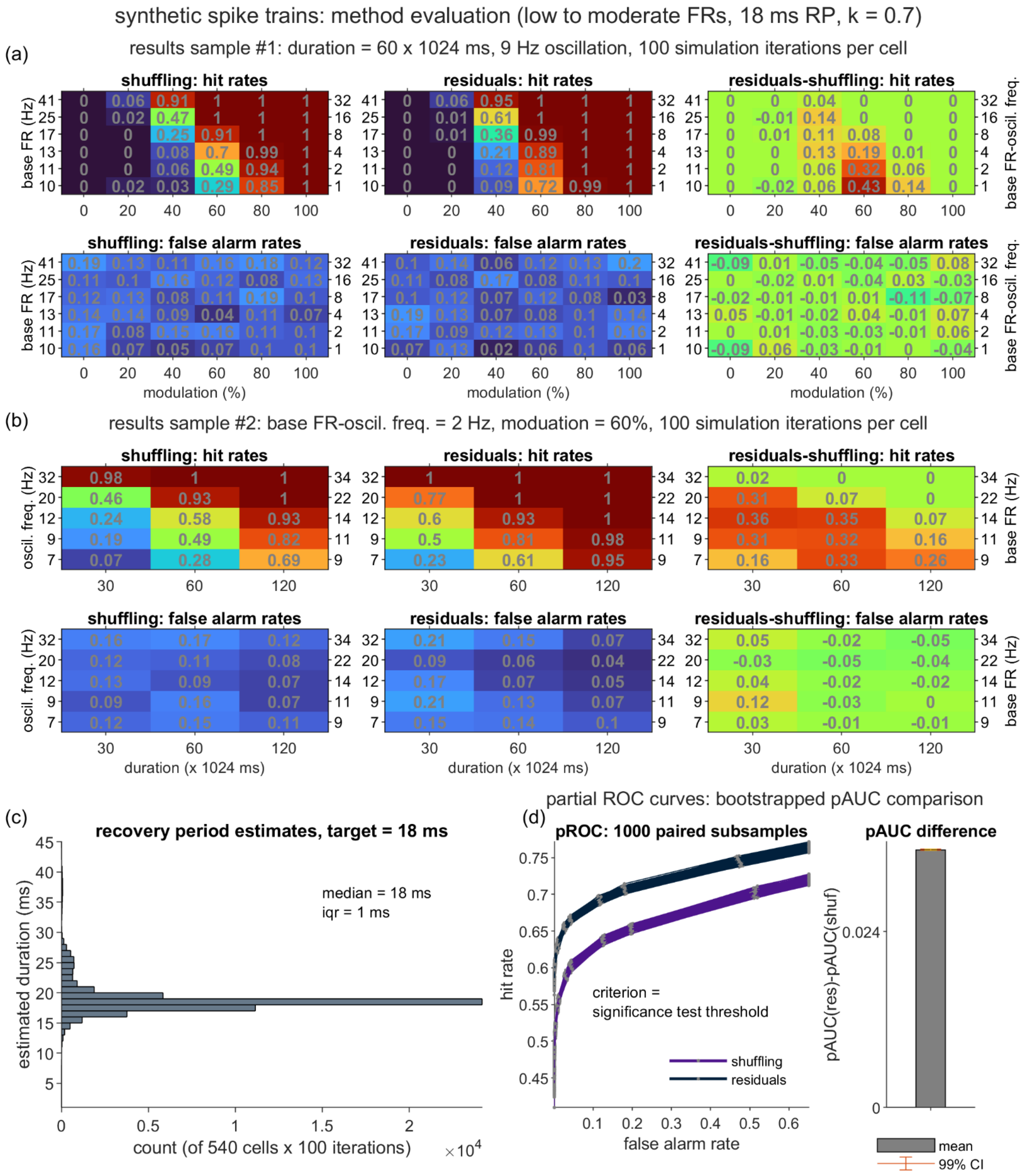

bioRxiv preprint doi: https://doi.org/10.1101/2023.09.08.556120; this version posted March 25, 2024. The copyright holder for this preprint (which was not certified by peer review) is the author/funder, who has granted bioRxiv a license to display the preprint in perpetuity. It is made available under aCC-BY 4.0 International license.

Fig. S14. Performance of the shuffling and residuals methods over a synthetic dataset generated using a lengthened relative recovery period. Panels (a)-(d): Plotting conventions, hit and false alarm definitions, and analysis procedures are identical to those described for the primary dataset depicted in Fig. 4. Relative to the primary dataset, this secondary dataset differed in the use of a longer relative RP (nr = 18 ms, k = 0.7).

bioRxiv preprint doi: https://doi.org/10.1101/2023.09.08.556120; this version posted March 25, 2024. The copyright holder for this preprint (which was not certified by peer review) is the author/funder, who has granted bioRxiv a license to display the preprint in perpetuity. It is made available under aCC-BY 4.0 International license.

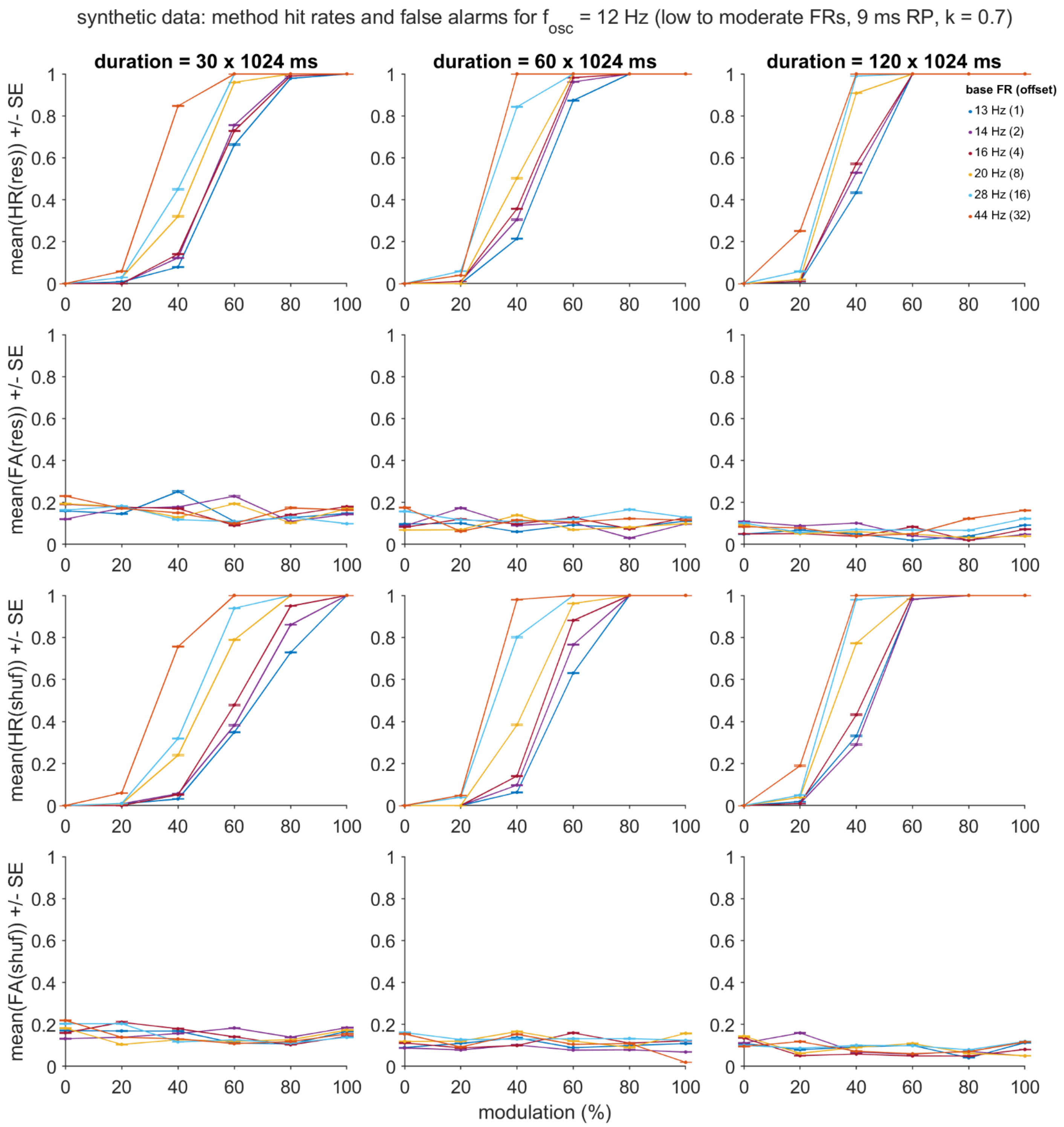

bioRxiv preprint doi: https://doi.org/10.1101/2023.09.08.556120; this version posted March 25, 2024. The copyright holder for this preprint (which was not certified by peer review) is the author/funder, who has granted bioRxiv a license to display the preprint in perpetuity. It is made available under aCC-BY 4.0 International license.

Fig. S15. Residuals and shuffling hit and false alarm rates for the primary synthetic dataset, with the oscillation frequency ($f_{osc}$) fixed at 12 Hz. Abbreviations and plotting conventions follow from those described for Fig. S9-S10.

bioRxiv preprint doi: https://doi.org/10.1101/2023.09.08.556120; this version posted March 25, 2024. The copyright holder for this preprint (which was not certified by peer review) is the author/funder, who has granted bioRxiv a license to display the preprint in perpetuity. It is made available under aCC-BY 4.0 International license.

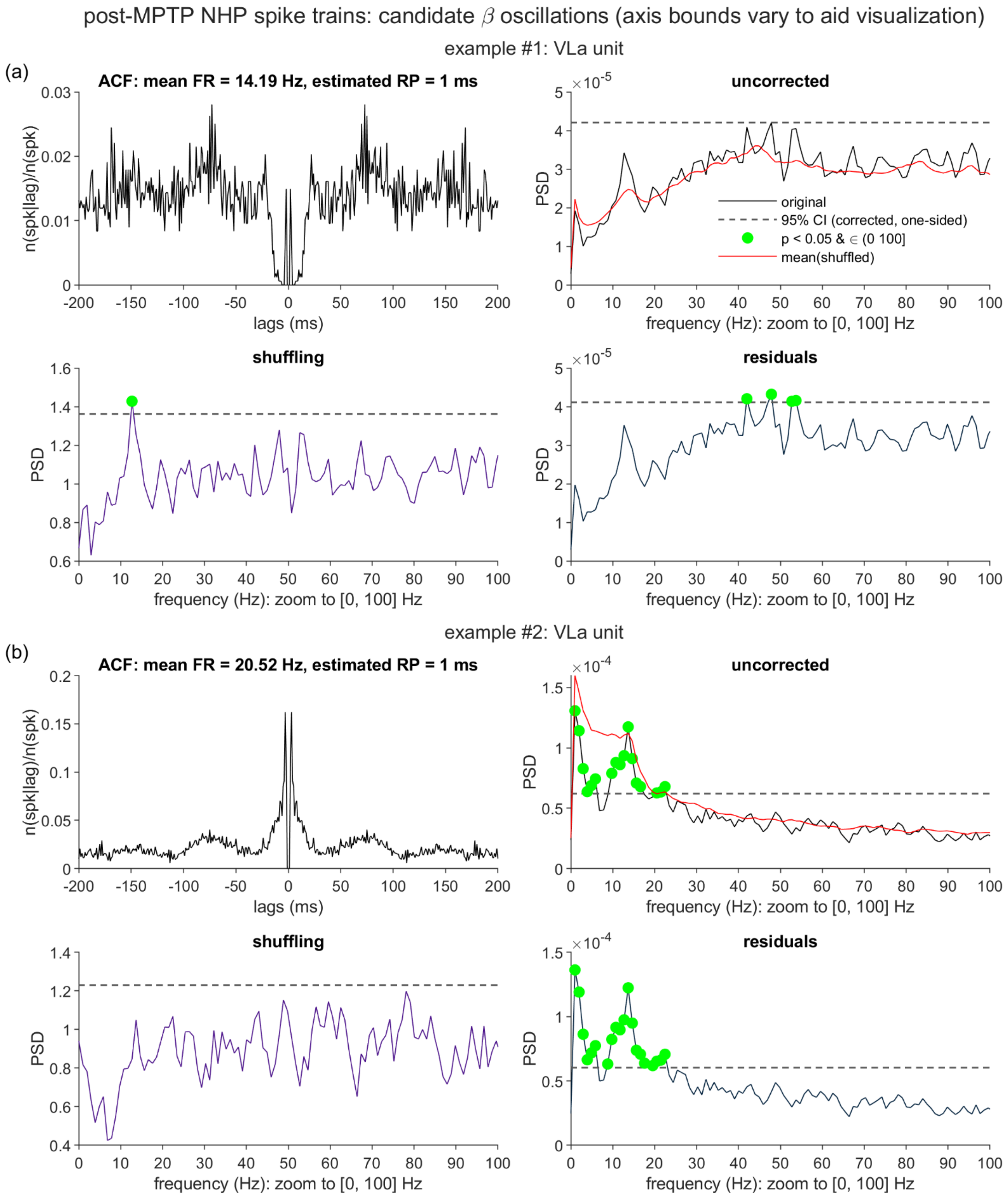

bioRxiv preprint doi: https://doi.org/10.1101/2023.09.08.556120; this version posted March 25, 2024. The copyright holder for this preprint (which was not certified by peer review) is the author/funder, who has granted bioRxiv a license to display the preprint in perpetuity. It is made available under aCC-BY 4.0 International license.

Fig. S16. Comparison of the shuffling and residuals output for two units with putative beta oscillations and non-oscillatory features. Spike trains originated from two ventrolateral anterior thalamus (VLa) units, which had been recorded from the same parkinsonian non-human primate (NHP) that contributed to Fig. 5-6. Panels (a)-(b): Abbreviations and the statistical and plotting conventions follow from those described for Fig. 5.

bioRxiv preprint doi: https://doi.org/10.1101/2023.09.08.556120; this version posted March 25, 2024. The copyright holder for this preprint (which was not certified by peer review) is the author/funder, who has granted bioRxiv a license to display the preprint in perpetuity. It is made available under aCC-BY 4.0 International license.

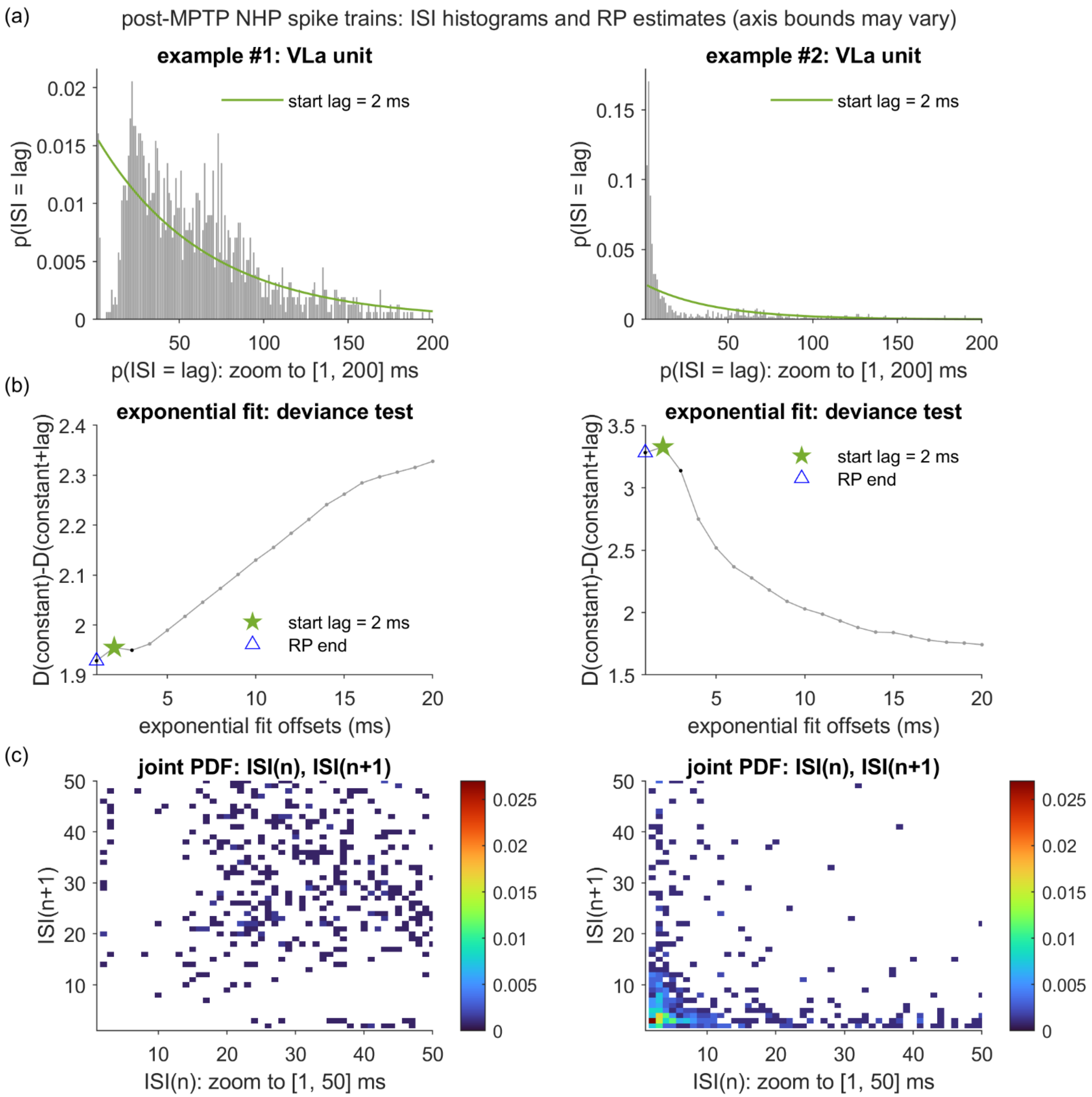

bioRxiv preprint doi: https://doi.org/10.1101/2023.09.08.556120; this version posted March 25, 2024. The copyright holder for this preprint (which was not certified by peer review) is the author/funder, who has granted bioRxiv a license to display the preprint in perpetuity. It is made available under aCC-BY 4.0 International license.

Fig. S17. Inter-spike interval distributions and RP duration estimation for the two VLa units with suspected oscillatory and non-oscillatory features. The left and right columns depict the same two empirical spike trains as were presented in Fig. S16(a)-(b). (a) Illustration of the procedure for obtaining the estimated RP duration ($\hat{n}_r$) for the two spike trains. See Fig. 3(a)-(b) and the Methods for full details. For each unit, the figure highlights the exponential curve with the left anchor position ("start lag") that returned the first local maximum in the curve goodness-of-fit plot. (b) Plots of the goodness-of-fit measure (the deviance difference statistic, ΔD) as a function of the first 20 starting positions of the exponential fits. Each $\hat{n}_r$ estimate (blue triangles) was set equal to the post-spike lag immediately preceding the first local maximum in the corresponding ΔD plot (green stars). (c) Joint probability density function (PDF) for all consecutive [$ISI_n$, $ISI_{n+1}$] inter-spike interval pairs. All remaining abbreviations follow from those described in Fig. 3 and Fig. 5.

bioRxiv preprint doi: https://doi.org/10.1101/2023.09.08.556120; this version posted March 25, 2024. The copyright holder for this preprint (which was not certified by peer review) is the author/funder, who has granted bioRxiv a license to display the preprint in perpetuity. It is made available under aCC-BY 4.0 International license.

## Supporting Tables

Table S1. Parametric Effects on Residuals-Shuffling Hit Rates
Low-to-Moderate Firing Rates (9 ms RP, k = 0.7)

| Effect | Beta | SE | t | p |
|---|---|---|---|---|
| Intercept | 0.084 | 0.00017 | 494.17 | -- |
| T | -0.00086 | 4.6e-06 | -188.48 | -- |
| $f_{osc}$ | -0.0049 | 1.9e-05 | -263.45 | -- |
| $p_{base\_offset}$ | -0.0046 | 1.6e-05 | -292.25 | -- |
| m | -0.17 | 0.0012 | -143.68 | -- |
| $m^2$ | -0.48 | 0.0016 | -296.41 | -- |
| T x $f_{osc}$ | 3.4e-05 | 5e-07 | 68.25 | -- |
| T x $p_{base\_offset}$ | 3.5e-05 | 4.2e-07 | 82.42 | -- |
| $f_{osc}$ x $p_{base\_offset}$ | 0.00027 | 1.7e-06 | 155.21 | -- |
| T x m | -0.0024 | 3.1e-05 | -77.28 | -- |
| $f_{osc}$ x m | -0.0028 | 0.00013 | -21.75 | 7.6e-105 |
| $p_{base\_offset}$ x m | 0.00057 | 0.00011 | 5.29 | 1.2e-07 |
| T x $m^2$ | 0.0049 | 4.4e-05 | 112.53 | -- |
| $f_{osc}$ x $m^2$ | 0.03 | 0.00018 | 168.36 | -- |
| $p_{base\_offset}$ x $m^2$ | 0.029 | 0.00015 | 191.37 | -- |
| m x $m^2$ | 1.1 | 0.0081 | 132.67 | -- |
| T x $f_{osc}$ x $p_{base\_offset}$ | -9.9e-07 | 3e-08 | -33.48 | 2e-245 |
| T x $f_{osc}$ x m | 8.4e-05 | 1.1e-06 | 73.92 | -- |
| T x $p_{base\_offset}$ x m | 6.4e-05 | 9.5e-07 | 66.88 | -- |
| $f_{osc}$ x $p_{base\_offset}$ x m | 0.00017 | 3.9e-06 | 44.01 | -- |
| T x $f_{osc}$ x $m^2$ | -0.00015 | 4.8e-06 | -32.06 | 3.3e-225 |
| T x $p_{base\_offset}$ x $m^2$ | -0.00016 | 4e-06 | -40.27 | -- |
| $f_{osc}$ x $p_{base\_offset}$ x $m^2$ | -0.0015 | 1.7e-05 | -90.54 | -- |
| T x m x $m^2$ | 0.013 | 0.00022 | 60.29 | -- |
| $f_{osc}$ x m x $m^2$ | 0.0075 | 0.00088 | 8.54 | 1.3e-17 |
| $p_{base\_offset}$ x m x $m^2$ | -0.012 | 0.00074 | -16.60 | 7e-62 |

RP = recovery period; T = duration;

$f_{osc}$ = oscillation frequency;

$p_{base\_offset}$ = offset of base firing rate from $f_{osc}$;
m = oscillation modulation
strength;
-- = outside range of double representation

bioRxiv preprint doi: https://doi.org/10.1101/2023.09.08.556120; this version posted March 25, 2024. The copyright holder for this preprint (which was not certified by peer review) is the author/funder, who has granted bioRxiv a license to display the preprint in perpetuity. It is made available under aCC-BY 4.0 International license.

Table S2. Parametric Effects on Residuals-Shuffling False Alarms
Low-to-Moderate Firing Rates (9 ms RP, k = 0.7)

| Effect | Beta | SE | t | p |
|---|---|---|---|---|
| Intercept | -0.023 | 0.00017 | -138.04 | -- |
| T | -0.00034 | 4.5e-06 | -75.60 | -- |
| $f_{osc}$ | -0.0012 | 1.8e-05 | -64.95 | -- |
| $p_{base\_offset}$ | -4.3e-05 | 1.5e-05 | -2.81 | 0.0049 |
| m | -0.0026 | 0.0011 | -2.28 | 0.023 |
| $m^2$ | 0.095 | 0.0016 | 59.43 | -- |
| T x $f_{osc}$ | 1.2e-05 | 4.9e-07 | 24.87 | 1.9e-136 |
| T x $p_{base\_offset}$ | 1.1e-05 | 4.1e-07 | 27.19 | 1.2e-162 |
| $f_{osc}$ x $p_{base\_offset}$ | 4.3e-06 | 1.7e-06 | 2.54 | 0.011 |
| T x m | 0.001 | 3e-05 | 34.38 | 9.8e-259 |
| $f_{osc}$ x m | -0.0031 | 0.00012 | -25.25 | 1.4e-140 |
| $p_{base\_offset}$ x m | 0.0055 | 0.0001 | 52.64 | -- |
| T x $m^2$ | 0.00053 | 4.3e-05 | 12.32 | 6.9e-35 |
| $f_{osc}$ x $m^2$ | -0.0027 | 0.00017 | -15.26 | 1.4e-52 |
| $p_{base\_offset}$ x $m^2$ | 0.0031 | 0.00015 | 21.28 | 2.1e-100 |
| m x $m^2$ | 0.031 | 0.0079 | 3.93 | 8.6e-05 |
| T x $f_{osc}$ x $p_{base\_offset}$ | -1e-06 | 2.9e-08 | -34.66 | 7.9e-263 |
| T x $f_{osc}$ x m | 2.3e-05 | 1.1e-06 | 20.65 | 1e-94 |
| T x $p_{base\_offset}$ x m | 2.1e-05 | 9.3e-07 | 22.26 | 9.5e-110 |
| $f_{osc}$ x $p_{base\_offset}$ x m | -0.00025 | 3.8e-06 | -65.86 | -- |
| T x $f_{osc}$ x $m^2$ | 2.1e-05 | 4.7e-06 | 4.39 | 1.1e-05 |
| T x $p_{base\_offset}$ x $m^2$ | -1.5e-05 | 3.9e-06 | -3.72 | 0.0002 |
| $f_{osc}$ x $p_{base\_offset}$ x $m^2$ | -0.00043 | 1.6e-05 | -26.54 | 4.8e-155 |
| T x m x $m^2$ | -0.0034 | 0.00021 | -16.24 | 2.8e-59 |
| $f_{osc}$ x m x $m^2$ | 0.0067 | 0.00086 | 7.73 | 1e-14 |
| $p_{base\_offset}$ x m x $m^2$ | -0.027 | 0.00073 | -37.01 | 2.5e-299 |

RP = recovery period; T = duration;

$f_{osc}$ = oscillation frequency;

$p_{base\_offset}$ = offset of base firing rate from $f_{osc}$;
m = oscillation modulation
strength;

-- = outside range of double representation

bioRxiv preprint doi: https://doi.org/10.1101/2023.09.08.556120; this version posted March 25, 2024. The copyright holder for this preprint (which was not certified by peer review) is the author/funder, who has granted bioRxiv a license to display the preprint in perpetuity. It is made available under aCC-BY 4.0 International license.

Table S3. Parametric Effects on Residuals-Shuffling Hit Rates

Low-to-Moderate Firing Rates (9 ms RP, k = 0.7)

| Effect | Beta | SE | t | p |
|---|---|---|---|---|
| Intercept | 0.1 | 0.0003 | 341.42 | -- |
| T | -0.00099 | 8e-06 | -124.45 | -- |
| $p_{base}$ | -0.0052 | 0.00012 | -43.21 | -- |
| $p_{base\_offset}$ | -0.008 | 0.00013 | -59.81 | -- |
| m | -0.44 | 0.002 | -217.82 | -- |
| $m^2$ | -0.62 | 0.0029 | -217.13 | -- |
| T x $p_{base}$ | 8.9e-05 | 3.2e-06 | 27.85 | 3.5e-170 |
| T x $p_{base\_offset}$ | 4e-05 | 3.6e-06 | 11.29 | 1.5e-29 |
| $p_{base}$ x $p_{base\_offset}$ | 0.00036 | 5.3e-05 | 6.66 | 2.7e-11 |
| T x m | -0.004 | 5.4e-05 | -72.62 | -- |
| $p_{base}$ x m | -0.018 | 0.00082 | -22.29 | 7.4e-110 |
| $p_{base\_offset}$ x m | 0.013 | 0.00091 | 14.57 | 5e-48 |
| T x $m^2$ | 0.0079 | 7.7e-05 | 103.32 | -- |
| $p_{base}$ x $m^2$ | 0.036 | 0.0011 | 31.79 | 6.4e-221 |
| $p_{base\_offset}$ x $m^2$ | 0.053 | 0.0013 | 41.28 | -- |
| m x $m^2$ | 2.7 | 0.014 | 193.84 | -- |
| T x $p_{base}$ x $p_{base\_offset}$ | -7.2e-06 | 9.2e-07 | -7.81 | 5.8e-15 |
| T x $p_{base}$ x m | 2.2e-05 | 7.3e-06 | 3.03 | 0.0025 |
| T x $p_{base\_offset}$ x m | 0.00023 | 8.1e-06 | 28.19 | 3e-174 |
| $p_{base}$ x $p_{base\_offset}$ x m | -4.1e-05 | 0.00012 | -0.34 | 0.74 |
| T x $p_{base}$ x $m^2$ | -0.00045 | 3.1e-05 | -14.64 | 1.8e-48 |
| T x $p_{base\_offset}$ x $m^2$ | -0.00062 | 3.4e-05 | -17.96 | 5.3e-72 |
| $p_{base}$ x $p_{base\_offset}$ x $m^2$ | -0.0019 | 0.00051 | -3.77 | 0.00016 |
| T x m x $m^2$ | 0.024 | 0.00038 | 64.06 | -- |
| $p_{base}$ x m x $m^2$ | 0.11 | 0.0057 | 20.26 | 4e-91 |
| $p_{base\_offset}$ x m x $m^2$ | -0.084 | 0.0063 | -13.21 | 7.7e-40 |

RP = recovery period; T = duration;

$p_{base}$ = base firing rate;

$p_{base\_offset}$ = offset of base firing rate from $f_{osc}$;

m = oscillation modulation strength;

-- = outside range of double representation

bioRxiv preprint doi: https://doi.org/10.1101/2023.09.08.556120; this version posted March 25, 2024. The copyright holder for this preprint (which was not certified by peer review) is the author/funder, who has granted bioRxiv a license to display the preprint in perpetuity. It is made available under aCC-BY 4.0 International license.

Table S4. Parametric Effects on Residuals-Shuffling False Alarms
Low-to-Moderate Firing Rates (9 ms RP, k = 0.7)

| Effect | Beta | SE | t | p |
|---|---|---|---|---|
| Intercept | -0.022 | 0.00033 | -67.70 | -- |
| T | -0.00039 | 8.8e-06 | -44.08 | -- |
| $p_{base}$ | -0.0039 | 0.00013 | -29.52 | 8.9e-191 |
| $p_{base\_offset}$ | -0.0036 | 0.00015 | -24.56 | 6.4e-133 |
| m | -0.018 | 0.0022 | -8.17 | 3e-16 |
| $m^2$ | 0.089 | 0.0032 | 28.06 | 1.2e-172 |
| T x $p_{base}$ | 3.1e-05 | 3.5e-06 | 8.67 | 4.4e-18 |
| T x $p_{base\_offset}$ | 1.7e-05 | 3.9e-06 | 4.23 | 2.3e-05 |
| $p_{base}$ x $p_{base\_offset}$ | 0.0019 | 5.9e-05 | 32.70 | 1.8e-233 |
| T x m | 0.0019 | 6e-05 | 32.31 | 4.4e-228 |
| $p_{base}$ x m | 0.019 | 0.0009 | 21.40 | 1.9e-101 |
| $p_{base\_offset}$ x m | -0.0023 | 0.001 | -2.30 | 0.021 |
| T x $m^2$ | 0.0011 | 8.4e-05 | 12.82 | 1.3e-37 |
| $p_{base}$ x $m^2$ | 0.026 | 0.0013 | 20.63 | 2e-94 |
| $p_{base\_offset}$ x $m^2$ | 0.022 | 0.0014 | 15.36 | 3.3e-53 |
| m x $m^2$ | 0.25 | 0.016 | 16.26 | 2.2e-59 |
| T x $p_{base}$ x $p_{base\_offset}$ | -1.1e-05 | 1e-06 | -10.86 | 1.9e-27 |
| T x $p_{base}$ x m | 6.7e-05 | 8e-06 | 8.43 | 3.5e-17 |
| T x $p_{base\_offset}$ x m | 0.00014 | 8.9e-06 | 15.81 | 3.2e-56 |
| $p_{base}$ x $p_{base\_offset}$ x m | 0.0015 | 0.00013 | 11.19 | 4.6e-29 |
| T x $p_{base}$ x $m^2$ | 9.3e-05 | 3.4e-05 | 2.74 | 0.0061 |
| T x $p_{base\_offset}$ x $m^2$ | -0.00022 | 3.8e-05 | -5.86 | 4.5e-09 |
| $p_{base}$ x $p_{base\_offset}$ x $m^2$ | -0.0058 | 0.00056 | -10.21 | 1.8e-24 |
| T x m x $m^2$ | -0.0095 | 0.00042 | -22.76 | 2.2e-114 |
| $p_{base}$ x m x $m^2$ | -0.11 | 0.0062 | -17.11 | 1.4e-65 |
| $p_{base\_offset}$ x m x $m^2$ | 0.054 | 0.007 | 7.78 | 7e-15 |

RP = recovery period; T = duration;

$p_{base}$ = base firing rate;

$p_{base\_offset}$ = offset of base firing rate from $f_{osc}$;

m = oscillation modulation strength;

-- = outside range of double representation

bioRxiv preprint doi: https://doi.org/10.1101/2023.09.08.556120; this version posted March 25, 2024. The copyright holder for this preprint (which was not certified by peer review) is the author/funder, who has granted bioRxiv a license to display the preprint in perpetuity. It is made available under aCC-BY 4.0 International license.

Table S5. Parametric Effects on Residuals-Shuffling Hit Rates

High Firing Rates (9 ms RP, k = 0.7)

| Effect | Beta | SE | t | p |
|---|---|---|---|---|
| Intercept | 0.0011 | 6.2e-05 | 18.48 | 3.4e-76 |
| T | -4.8e-05 | 1.7e-06 | -28.85 | 6.6e-183 |
| $f_{osc}$ | -0.00011 | 6.8e-06 | -15.83 | 1.9e-56 |
| $p_{base\_offset}$ | -0.00019 | 3.6e-06 | -51.56 | -- |
| m | -0.018 | 0.00042 | -42.45 | -- |
| $m^2$ | 0.045 | 0.00059 | 75.82 | -- |
| T x $f_{osc}$ | 2.6e-06 | 1.8e-07 | 14.60 | 2.8e-48 |
| T x $p_{base\_offset}$ | 5.1e-06 | 9.7e-08 | 52.69 | -- |
| $f_{osc}$ x $p_{base\_offset}$ | 5.1e-06 | 4e-07 | 12.96 | 2.2e-38 |
| T x m | 0.00056 | 1.1e-05 | 49.35 | -- |
| $f_{osc}$ x m | 0.00094 | 4.6e-05 | 20.44 | 8.3e-93 |
| $p_{base\_offset}$ x m | 0.0017 | 2.5e-05 | 69.09 | -- |
| T x $m^2$ | -0.00045 | 1.6e-05 | -28.05 | 5.7e-173 |
| $f_{osc}$ x $m^2$ | 0.0013 | 6.5e-05 | 20.03 | 3e-89 |
| $p_{base\_offset}$ x $m^2$ | 0.0018 | 3.5e-05 | 51.76 | -- |
| m x $m^2$ | -0.015 | 0.0029 | -5.27 | 1.4e-07 |
| T x $f_{osc}$ x $p_{base\_offset}$ | -1.5e-08 | 6.8e-09 | -2.22 | 0.026 |
| T x $f_{osc}$ x m | 5.5e-08 | 4.1e-07 | 0.13 | 0.89 |
| T x $p_{base\_offset}$ x m | 4.5e-06 | 2.2e-07 | 20.60 | 3.3e-94 |
| $f_{osc}$ x $p_{base\_offset}$ x m | -7.2e-06 | 9e-07 | -8.00 | 1.3e-15 |
| T x $f_{osc}$ x $m^2$ | -2.5e-05 | 1.7e-06 | -14.19 | 1.1e-45 |
| T x $p_{base\_offset}$ x $m^2$ | -6e-05 | 9.3e-07 | -64.42 | -- |
| $f_{osc}$ x $p_{base\_offset}$ x $m^2$ | -2.8e-05 | 3.8e-06 | -7.26 | 4e-13 |
| T x m x $m^2$ | -0.0017 | 7.8e-05 | -22.31 | 3.2e-110 |
| $f_{osc}$ x m x $m^2$ | -0.0078 | 0.00032 | -24.22 | 1.6e-129 |
| $p_{base\_offset}$ x m x $m^2$ | -0.013 | 0.00017 | -74.32 | -- |

RP = recovery period; T = duration;

$f_{osc}$ = oscillation frequency;

$p_{base\_offset}$ = offset of base firing rate from $f_{osc}$;

m = oscillation modulation strength;

-- = outside range of double representation

bioRxiv preprint doi: https://doi.org/10.1101/2023.09.08.556120; this version posted March 25, 2024. The copyright holder for this preprint (which was not certified by peer review) is the author/funder, who has granted bioRxiv a license to display the preprint in perpetuity. It is made available under aCC-BY 4.0 International license.

Table S6. Parametric Effects on Residuals-Shuffling False Alarms
High Firing Rates (9 ms RP, k = 0.7)

| Effect | Beta | SE | t | p |
|---|---|---|---|---|
| Intercept | -0.014 | 0.00022 | -63.65 | -- |
| T | 1.5e-05 | 6e-06 | 2.53 | 0.011 |
| $f_{osc}$ | -0.00052 | 2.4e-05 | -21.37 | 3e-101 |
| $p_{base\_offset}$ | 0.00024 | 1.3e-05 | 18.24 | 2.5e-74 |
| m | 0.12 | 0.0015 | 76.88 | -- |
| $m^2$ | 0.23 | 0.0021 | 107.57 | -- |
| T x $f_{osc}$ | -5.4e-06 | 6.5e-07 | -8.25 | 1.6e-16 |
| T x $p_{base\_offset}$ | -6.7e-06 | 3.5e-07 | -19.16 | 8.6e-82 |
| $f_{osc}$ x $p_{base\_offset}$ | 1.3e-05 | 1.4e-06 | 9.33 | 1e-20 |
| T x m | 4.2e-05 | 4.1e-05 | 1.04 | 0.3 |
| $f_{osc}$ x m | -0.0089 | 0.00017 | -53.48 | -- |
| $p_{base\_offset}$ x m | 0.002 | 8.9e-05 | 22.75 | 1.6e-114 |
| T x $m^2$ | 0.0005 | 5.7e-05 | 8.83 | 1e-18 |
| $f_{osc}$ x $m^2$ | -0.079 | 0.00023 | -336.62 | -- |
| $p_{base\_offset}$ x $m^2$ | 0.0057 | 0.00013 | 45.72 | -- |
| m x $m^2$ | -0.11 | 0.011 | -10.32 | 5.9e-25 |
| T x $f_{osc}$ x $p_{base\_offset}$ | -6.5e-07 | 2.4e-08 | -26.43 | 7.2e-154 |
| T x $f_{osc}$ x m | -0.00026 | 1.5e-06 | -178.70 | -- |
| T x $p_{base\_offset}$ x m | 2.8e-05 | 7.9e-07 | 35.75 | 1.9e-279 |
| $f_{osc}$ x $p_{base\_offset}$ x m | -0.00059 | 3.2e-06 | -181.70 | -- |
| T x $f_{osc}$ x $m^2$ | -0.00069 | 6.2e-06 | -110.94 | -- |
| T x $p_{base\_offset}$ x $m^2$ | 0.00015 | 3.3e-06 | 45.24 | -- |
| $f_{osc}$ x $p_{base\_offset}$ x $m^2$ | -0.0016 | 1.4e-05 | -116.58 | -- |
| T x m x $m^2$ | 0.006 | 0.00028 | 21.19 | 1.3e-99 |
| $f_{osc}$ x m x $m^2$ | -0.15 | 0.0012 | -129.27 | -- |
| $p_{base\_offset}$ x m x $m^2$ | 0.0062 | 0.00062 | 10.05 | 9.2e-24 |

RP = recovery period; T = duration;

$f_{osc}$ = oscillation frequency;

$p_{base\_offset}$ = offset of base firing rate from $f_{osc}$;
m = oscillation modulation
strength;

-- = outside range of double representation
